# Identifying Adolescent Depression and Anxiety Through Real-World Data and Social Determinants of Health: Machine Learning Model Development and Validation

**DOI:** 10.2196/66665

**Published:** 2025-02-12

**Authors:** Mamoun T Mardini, Georges E Khalil, Chen Bai, Aparna Menon DivaKaran, Jessica M Ray

**Affiliations:** 1 Department of Health Outcomes and Biomedical Informatics, College of Medicine, University of Florida, 7th Floor, Suite 7000, 1889 Museum Rd, Gainesville, FL, 32611, United States, 1 7049045847

**Keywords:** social determinants of health, adolescents, anxiety, depression, machine learning, real-world data, teenagers, youth, XGBoost, cross-validation technique, SHapley Additive exPlanation, mental health, mental disorder, mental illness, health outcomes, clinical data

## Abstract

**Background:**

The prevalence of adolescent mental health conditions such as depression and anxiety has significantly increased. Despite the potential of machine learning (ML), there is a shortage of models that use real-world data (RWD) to enhance early detection and intervention for these conditions.

**Objective:**

This study aimed to identify depression and anxiety in adolescents using ML techniques on RWD and social determinants of health (SDoH).

**Methods:**

We analyzed RWD of adolescents aged 10‐17 years, considering various factors such as demographics, prior diagnoses, prescribed medications, medical procedures, and laboratory measurements recorded before the onset of anxiety or depression. Clinical data were linked with SDoH at the block-level. Three separate models were developed to predict anxiety, depression, and both conditions. Our ML model of choice was Extreme Gradient Boosting (XGBoost) and we evaluated its performance using the nested cross-validation technique. To interpret the model predictions, we used the Shapley additive explanation method.

**Results:**

Our cohort included 52,054 adolescents, identifying 12,572 with anxiety, 7812 with depression, and 14,019 with either condition. The models achieved area under the curve values of 0.80 for anxiety, 0.81 for depression, and 0.78 for both combined. Excluding SDoH data had a minimal impact on model performance. Shapley additive explanation analysis identified gender, race, educational attainment, and various medical factors as key predictors of anxiety and depression.

**Conclusions:**

This study highlights the potential of ML in early identification of depression and anxiety in adolescents using RWD. By leveraging RWD, health care providers may more precisely identify at-risk adolescents and intervene earlier, potentially leading to improved mental health outcomes.

## Introduction

The United States is experiencing a national health emergency as rates of childhood and adolescent mental health conditions, including depression and anxiety, doubled during the COVID-19 pandemic [1,2]. An estimated 49.5% of individuals experience a mental health concern in their lifetime, and nearly half of those cases occur by the age of 14 [3,4]. Mental illnesses with significant impairment affect approximately 1 in 4 adolescents [5]. Consequently, new and innovative strategies for early detection are critical [6]. However, early detection and access to early intervention are often limited due to a lack of specialized knowledge and resources [6]. The United States alone has an estimated deficit of 4.5 million trained mental health clinicians [6]. As a result, about half of the adolescents with mental health problems do not obtain mental health services despite having treatable conditions. Given the shortage of mental health specialists, primary care clinicians are a critical access point for screening and early treatment of pediatric mental health conditions [7]. This increased demand for mental health care, along with the already limited time available in routine primary care visits, creates the need for innovative approaches to support longitudinal care beyond the clinic.

Machine learning (ML) in health care provides flexibility and scalability in assimilating and evaluating large volumes of complex data, including those found in electronic health records (EHR) [8]. The EHRs span multiple domains beyond clinical tests and diagnoses, including social determinants of health (SDoH) such as education, economic circumstances, and social environment, all of which can significantly impact adolescents’ mental health and well-being [9-12]. Growing evidence suggests that ML algorithms can facilitate the early detection of symptoms such as depression [13]. Incorporating SDoH data into the predictive models for depression and anxiety in adolescents can potentially identify those who are at higher risk, which can have a significant impact on the practice of medicine by providing more accurate diagnoses and timely interventions.

Previous research has shown that SDoH are significantly associated with adolescent mental health outcomes [14]. Factors such as socioeconomic status, education, neighborhood environment, and access to health care contribute to disparities that impact mental well-being. Adolescents from low-income families are often affected by stressful environments and limited access to mental health services, driving increased rates of anxiety and depression [15-19]. In addition, educational environments can play a critical role; schools with abundant resources can promote better mental health among students [20]. Studies have also indicated that community factors such as social support and community engagement can improve resilience and mitigate adverse mental health outcomes. For instance, adolescents who experience a sense of belonging and trust within their community are less likely to experience severe mental health issues [21].

Despite this established association between SDoH and adolescent mental health, further research is needed to develop and improve ML models that effectively incorporate these SDoH factors. In the past, mental health research has mainly relied on questionnaires and neuroimaging data. While the use of EHR data shows promise, it has not been widely used in this area of research. This gap emphasizes the necessity for further exploration and innovation in leveraging EHR data to improve the accuracy of ML-driven mental health models for children.

## Methods

### Study Population and Outcome Variables

This retrospective research study used data from the University of Florida Integrated Data Repository (UF-IDR); it included only adolescents aged 10‐17 years. Patients diagnosed with anxiety or depression were identified using the *ICD-10* (*International Statistical Classification of Disease and Related Health Problems, 10th Revision*), as shown in Table 1. Controls were matched to targeted cases based on age and sex in a ratio of 3:1.

**Table 1.** Description of *ICD-10* codes used to identify the target cohort.

*ICD-10* codes	Description
*F32*	Major depressive disorder, single episode
*F33*	Major depressive disorder, recurrent
*F34*	Persistent mood disorders
*F40-48*	Anxiety, dissociative, stress-related, somatoform, and other nonpsychotic mental disorders
*F93*	Emotional disorders with onset specific to childhood

### Ethical Considerations

The study was reviewed and approved by the Institutional Review Board of the University of Florida (IRB202301144). The study used existing data for secondary analysis, and primary consent was obtained previously. Therefore, additional consent was not obtained in this case. The data was deidentified and the participants were not compensated in any manner.

### Predictive Features

Predictive features in this study encompassed a variety of factors, including demographics, prior diagnoses, prescribed medications, medical procedures, and laboratory measurements recorded before the first episode of anxiety or depression. Additionally, SDoH features were incorporated from the National Historical Geographic Information System. These block-level SDoH features included employment rate, poverty level, educational attainment, health insurance coverage, household computer and internet access, and median household income. A comprehensive list of features is provided in Table S1 in Multimedia Appendix 1.

### Area Deprivation Index

The area deprivation index (ADI) assesses community deprivation and its impact on health outcomes and helps develop policies and health care utilization strategies [22]. Studies have shown that living in a disadvantaged area may have negative effects on health similar to certain chronic diseases [22]. The Centers for Medicare & Medicaid Services incorporate the ADI in their strategies. In this study, we used the ADI to examine community-level disparities. This index is calculated at the US Census block group level and represents neighborhoods of approximately 6003000 residents. This level of detail provides a localized view of health-related social and geospatial determinants [23]. We assigned ADI ranks to patient addresses based on their Florida residential Census block group, sourced from their 9-digit ZIP codes. Higher ADI ranks indicate greater social disadvantage.

### Data Linkage

To integrate EHR data from the UF-IDR with Census data from National Historical Geographic Information System, we followed a systematic process that used geographic identifiers (GEOIDs). First, we used patient addresses from the UF-IDR to acquire geographic coordinates (ie, latitude and longitude). These coordinates were then matched to Census block groups using GEOIDs that are unique numeric codes assigned to all geographic areas tabulated by the Census Bureau. Block Groups are divisions of Census tracts, typically containing 600-3000 people and are identified by the first digit of their four-digit Census block number. To merge the EHR and Census datasets, we made use of GEOIDs. By using the RStudio software (version 2024.12.0+467; R Foundation for Statistical Computing), *tigris* package (version 2.1), the function “call_geolocator_latlon’” converted latitude and longitude into corresponding Census tracts and blocks, providing the necessary GEOID. This GEOID then enabled us to combine patient data with Census block group data; each patient ID was associated with pertinent Census information. The ADI were linked at the block group level using Federal Information Processing Standards codes, equivalent to GEOIDs. This integrated dataset allowed for comprehensive analysis by merging detailed demographic and socioeconomic data from Census blocks with patient health records.

### Data Processing

We transformed the original codes of patients’ diagnoses and medical procedures into clinically meaningful categories using the Clinical Classification Software; only diagnoses, procedures, and medications present in at least 5% of patients were considered. Categorical variables were encoded to facilitate their inclusion in the analysis, and numerical variables were scaled using a standard scaler to ensure consistency across different scales. We used k-nearest neighbors imputation to address missing data. The SDoH variables had a maximum of 7% missingness per block group, while vital signs data had a substantial 20% missing data rate. We excluded patients with more than 50% missing SDoH variables or those without mapped ADI, resulting in a final dataset of 52,054 patients. Outliers in laboratory values were identified using the IQR method, which is calculated as the difference between the third quartile and the first quartile , was used to define outliers as values below Q1 - 1.5 * IQR or above Q3+1.5 * IQR. These identified outliers were replaced with the median value of the respective laboratory test, as the median is less sensitive to extreme values and provides a more robust measure of central tendency.

### Feature Selection

We followed a three-phase feature selection approach to eliminate features that did not significantly contribute to predicting the outcomes. First, we examined the association between each predictor and the target outcome using *χ*^2^ test for categorical features and *t*-test or Wilcoxon rank-sum test for continuous features, depending on normality assumptions. Features that did not exhibit significant differences between the two groups were eliminated. Second, we calculated pair-wise correlations between features and removed highly correlated ones (*r*>0.7) to mitigate multicollinearity. Finally, we performed the least absolute shrinkage and selection operator regression to further reduce this feature space. We examined different regularization strengths and selected the one that achieved the highest area under the receiver operating characteristic curve (AUC) for predicting the outcome.

### Machine Learning Model Development

We used Extreme Gradient Boosting (XGBoost), a robust ML framework known for its efficiency, flexibility, and portability [24]. It is an ensemble learning algorithm based on the gradient boosting framework, in which models are built sequentially to boost (ie, increase) the performance of the previous models by using the gradient descent algorithm to minimize errors [24]. Our selection of XGBoost is based on simplified interpretability and the inclusion of feature selection as part of the model-building process. XGBoost exhibits various advantages that make it a compelling alternative to conventional statistical techniques and other ML algorithms.

To address our research objectives, we developed 9 ML models based on the outcome and the type of predictor included. The first 3 models used all features to predict depression, anxiety, and both anxiety and depression together. The next three models replaced individual SDoH features with the ADI as a single representation of SDoH. The final three models conducted a sensitivity analysis by excluding SDoH entirely to evaluate its impact on predicting anxiety and depression and to assess its effect on the performance of the models for these mental health conditions.

### Model Performance and Evaluation

We used a 5×5 nested cross-validation technique to evaluate our ML models. This technique consisted of 5 outer and 5 inner folds. For each outer fold, we set aside one-fifth of the patient records as an independent testing set, while the remaining four-fifths formed the training set. This outer training subset was then split into 5 inner folds for further validation. Each inner fold served as a standalone validation set, while the other 4 functioned as the training set for the inner loop. The inner loop was responsible for training the models and fine-tuning the hyperparameters, using a methodical approach while searching for the model’s ideal hyperparameter settings. Meanwhile, the outer loop estimated errors and evaluated generalization capabilities. To optimize hyperparameters, we used a grid search strategy to systematically explore various combinations of predefined hyperparameters to train our models. We calculated and reported the mean and standard deviation for metrics such as AUC, accuracy, sensitivity, and specificity across the five outer folds. This thorough approach boosts confidence in the models’ generalizability and scalability.

### Model Interpretation and Feature Ranking

We used the Shapley additive explanation (SHAP) method to explain the functioning of our trained ML models. The SHAP is a widely used model-agnostic explanatory approach that helps in understanding the outputs of the ML model. We created a SHAP summary plot to demonstrate the importance of features and their impact on the outcome. This impact is shown through a sign and magnitude, where the SHAP value’s sign indicates the direction of the feature’s impact on the outcome (eg, a positive SHAP value indicates that the feature in question increases the likelihood of frailty), while its magnitude reflects the feature’s predictive influence.

## Results

The study encompassed a total of 52,054 patients, among whom a subset exhibited symptoms of anxiety, depression, or both conditions concurrently. Out of the total cohort, 12,572 patients were identified as experiencing anxiety, 7812 patients were diagnosed with depression, and 14,019 patients presented with anxiety or depression. Using this dataset, 3 distinct models were trained for each condition: anxiety, depression, and anxiety or depression.

Table 2 shows the predictive performance of the ML models. The model predicting both depression and anxiety achieved an AUC of 0.78. The model focusing on depression alone showed an AUC of 0.81, and the model concentrating on anxiety alone recorded an AUC of 0.80. The models built using only the ADI achieved similar performance, as shown in Table 3. When excluding SDoH, the performance of the ML models remained largely unaffected, as shown in Table 4. The model predicting both depression and anxiety achieved the same AUC of 0.78, the depression model maintained the AUC of 0.81. However, the performance of the anxiety model alone slightly decreased to an AUC of 0.78.

A sensitivity analysis was conducted by removing demographic features from the model for predicting the composite outcomes while incorporating SDoH features. The model achieved a slight decline in performance with a mean accuracy of 0.71, balanced accuracy of 0.67 , sensitivity of 0.60 (SD 0.01), specificity of 0.75 (SD 0.01), and AUC of 0.75 (SD 0.01).

**Figure 1.**
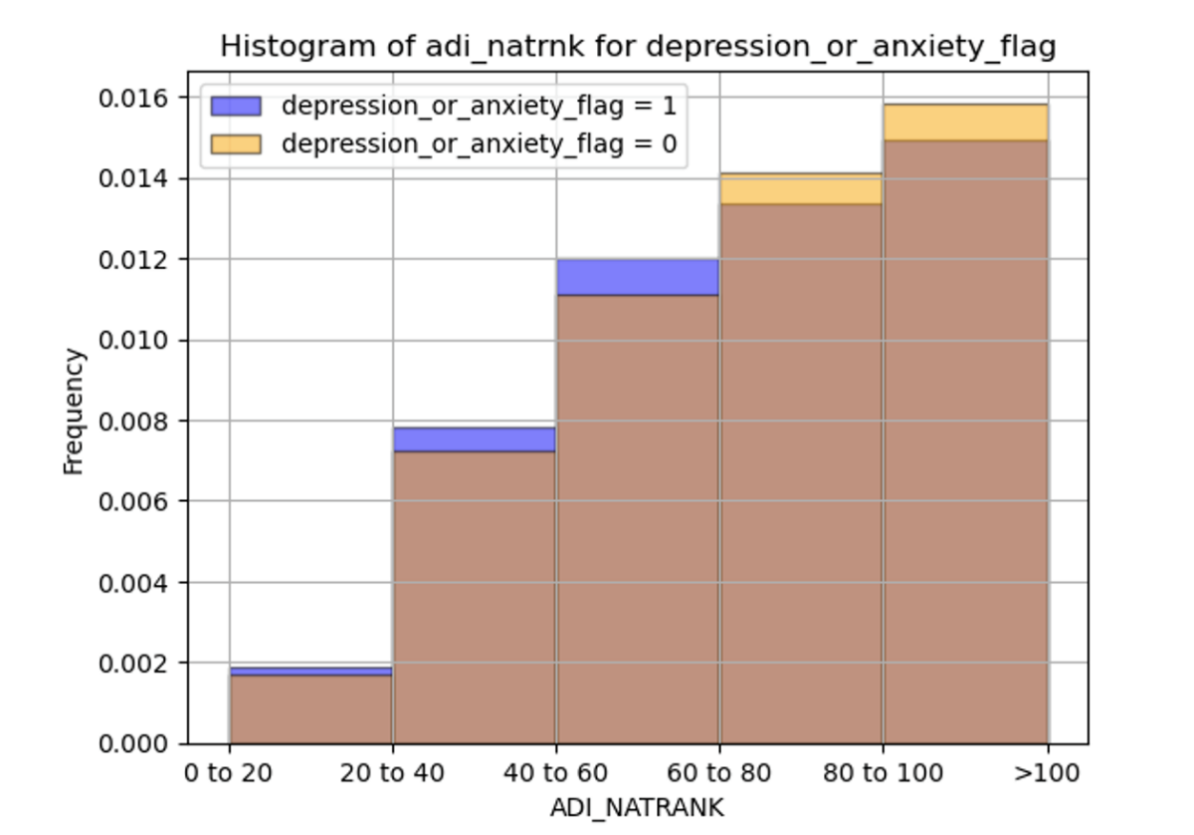
Distribution of anxiety and depression with respect to area deprivation index (ADI). ADI_NATRANK represents the national rank ranging from 0 (advantaged) to 100 (disadvantaged).

Figure 1 shows the distribution of anxiety and depression with respect to ADI. In the lower (advantaged) ADI national rank bins (0-20 and 20-40), the frequency of individuals with depression or anxiety is higher than those without these conditions. As the rank increases (40-60, 60-80, and 80-100), the frequency of both groups increases; however, the proportion of individuals without depression or anxiety becomes more significant. The highest (disadvantaged) ADI national rank bin (80-100) shows the largest frequencies for both groups, with a higher representation of individuals without depression or anxiety.

**Figure 2.**
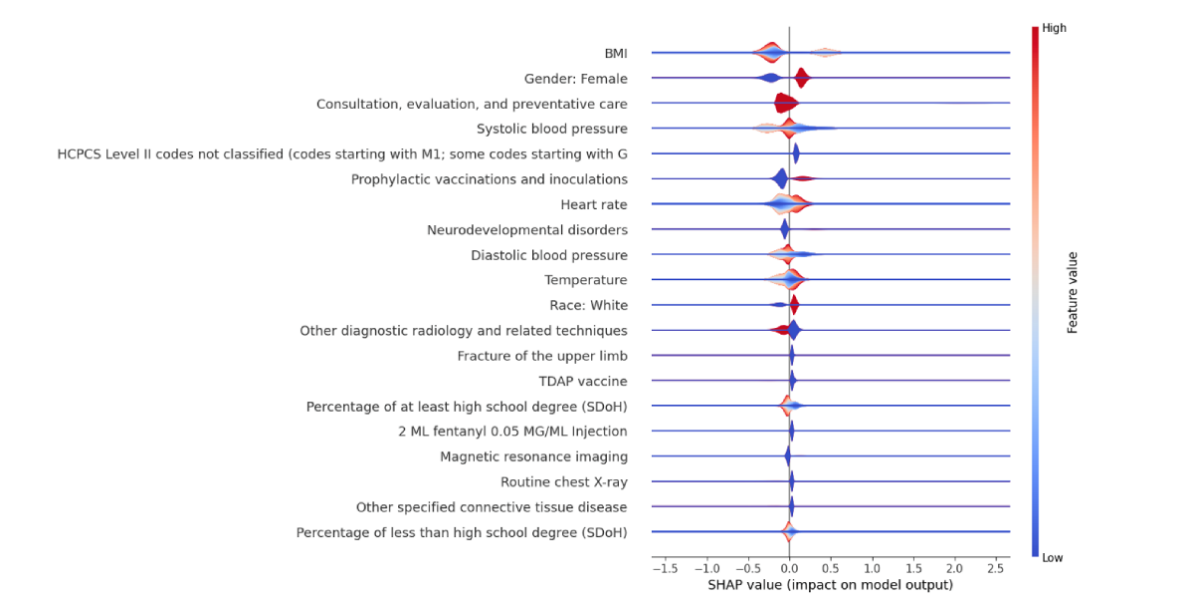
SHAP summary plot for predicting the combined outcome - depression or anxiety by incorporating SDoH features. SDoH: social determinants of health; SHAP: Shapley additive explanation; TDAP: tetanus, diphtheria, and pertussis.

**Figure 3.**
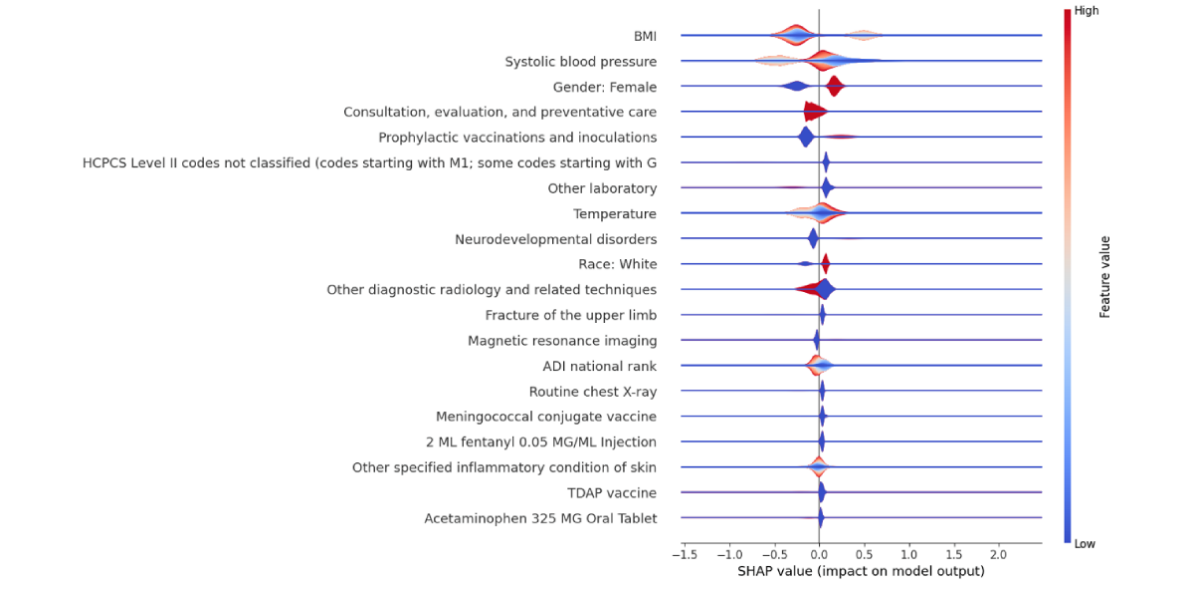
SHAP summary plot for predicting the combined outcome - depression or anxiety by incorporating only ADI. ADI: area deprivation index; SHAP: Shapley additive explanation; TDAP: tetanus, diphtheria, and pertussis.

Figure 2 shows the SHAP summary plot, which identifies various important features contributing to the prediction of anxiety and depression in adolescents. Key predictors include gender (with females being more vulnerable), race (showing a higher likelihood in White adolescents), and educational attainment (both high school diploma or General Educational Development and less than high school education). Additionally, medical indicators such as BMI, systolic and diastolic blood pressure, heart rate, and temperature, along with procedural and diagnostic factors, were found to be influential. Notably, adolescents with neurodevelopmental disorders or connective tissue diseases, along with those undergoing frequent diagnostic imaging or specific vaccinations, displayed a higher propensity for anxiety and depression. Figure 3 shows the SHAP summary plot for the model predicting both depression and anxiety while replacing the individual SDoH features with the ADI, which ranked 14th in importance among other features.

**Figure 4.**
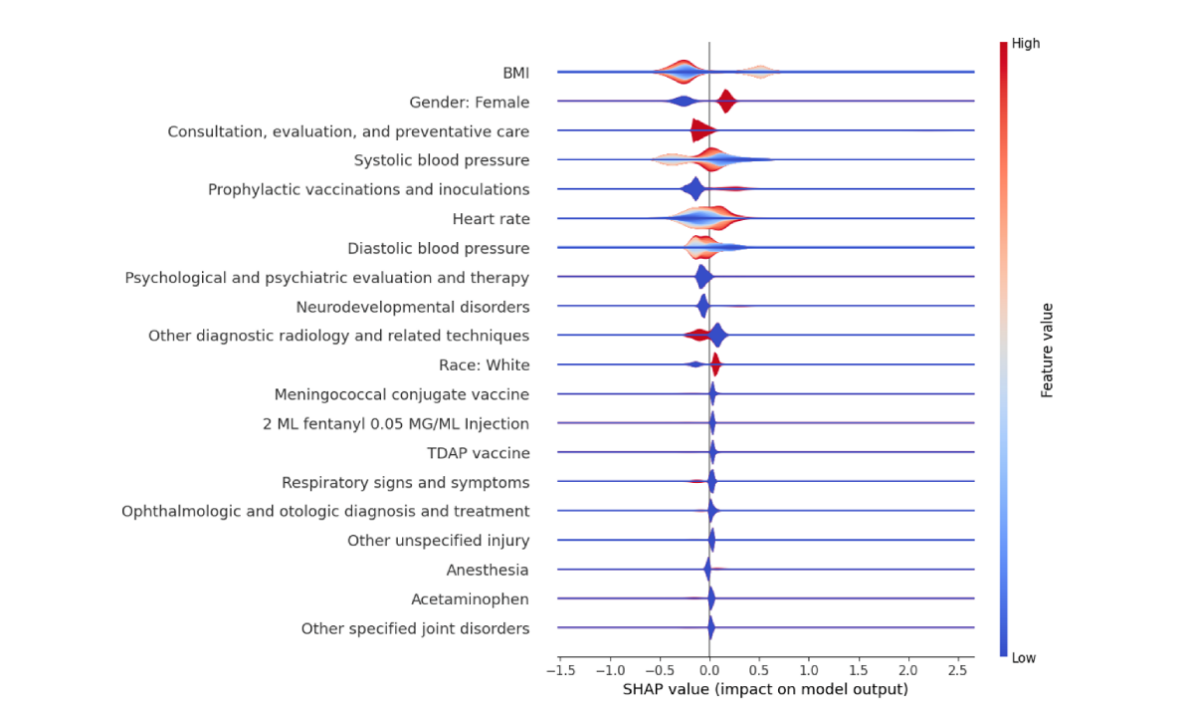
SHAP summary plot for predicting the combined outcome - depression or anxiety without incorporating SDoH features. SHAP: Shapley additive explanation; TDAP: tetanus, diphtheria, and pertussis.

Figure 4 presents the SHAP summary plot for the ML model, which combines both depression and anxiety while excluding SDoH predictors. Overall, there were slight changes in the list of important predictors. Additionally, Figures S1-S3 in Multimedia Appendix 1 display the SHAP summary plots for the individual ML models predicting depression with SDoH, ADI only, and without SDoH, respectively. Figures S4-S6 in Multimedia Appendix 1 display SHAP summary plots for the individual ML models predicting anxiety with SDoH, ADI only, and without SDoH, respectively.

**Table 2.** Predictive performance of ML models (presented as mean, SD) by incorporating SDoH features. The reported performance is presented using mean and standard deviation from the five outer folds.

	Accuracy, mean (SD)	Balanced accuracy, mean (SD)	Sensitivity, mean (SD)	Specificity, mean (SD)	AUC,^a^ mean (SD)
Depression and anxiety	0.73 (0)	0.70 (0.01)	0.65 (0.01)	0.75 (0.01)	0.78 (0)
Depression	0.76 (0.01)	0.73 (0)	0.68 (0.02)	0.78 (0.01)	0.81 (0.01)
Anxiety	0.75 (0.01)	0.72 (0)	0.66 (0.02)	0.78 (0.01)	0.80 (0)

a AUC: area under the curve.

**Table 3.** Predictive performance of ML models by incorporating ADI only. The reported performance is presented using mean and standard deviation from the five outer folds.

	Accuracy, mean (SD)	Balanced accuracy, mean (SD)	Sensitivity, mean (SD)	Specificity, mean (SD)	AUC,^a^ mean (SD)
Depression and anxiety	0.72 (0.01)	0.70 (0.01)	0.66 (0.01)	0.74 (0.01)	0.78 (0)
Depression	0.76 (0)	0.73 (0)	0.68 (0)	0.77 (0)	0.81 (0)
Anxiety	0.75 (0.01)	0.71 (0.01)	0.65 (0.01)	0.78 (0.01)	0.79 (0)

a AUC: area under the curve.

**Table 4.** Predictive performance of ML models without incorporating SDoH features. The reported performance is presented using mean and standard deviation from the five outer folds.

	Accuracy, mean (SD)	Balanced accuracy, mean (SD)	Sensitivity, mean (SD)	Specificity, mean (SD)	AUC,^a^ mean (SD)
Depression and anxiety	0.73 (0)	0.70 (0)	0.64 (0.01)	0.76 (0)	0.78 (0)
Depression	0.76 (0.01)	0.73 (0)	0.68 (0)	0.77 (0.01)	0.81 (0)
Anxiety	0.72 (0)	0.71 (0)	0.69 (0.01)	0.73 (0.01)	0.78 (0)

a AUC: area under the curve.

## Discussion

### Principal findings

Our study findings show that incorporating SDoH features, or ADI did not improve the predictive accuracy of the ML models for depression, anxiety, or a combination of both. While SDoH factors influence individuals’ health, their contribution to the model’s predictive performance was limited. Individuals with depression or anxiety were more prevalent in areas with lower (advantaged) ADI rankings. In contrast, in higher (disadvantaged) ADI areas, the overall number of individuals with and without mental health conditions increased, but the proportion of individuals without depression or anxiety was greater. This suggests that area deprivation is associated with mental health issues but is not a sole determinant. The lack of improvement in model performance with the inclusion of SDoH features may be attributed to using community-level data, which does not account for individual differences. Future studies should explore individual-level SDoH data, potentially extracted from clinical notes, to improve the detail and predictive accuracy of ML models.

The results emphasize the significant impact of various factors on adolescents’ mental health. The higher prevalence of anxiety and depression among female and White adolescents may reflect the influence of social, cultural, and systemic stressors. For women, societal pressures, gender-based expectations, and hormonal factors may contribute to higher rates of anxiety and depression. For White adolescents, the observed prevalence may be linked to differential access to mental health diagnoses or treatment, health care–seeking behaviors, or unmeasured cultural stressors. Future studies should explore these patterns in detail, accounting for intersectionality and regional differences. The strong effect of educational attainment highlights the importance of supportive educational environments in reducing mental health risks. Medical factors including heart rate, systolic and diastolic blood pressure, and temperature were notable predictors. These physiological measures may indicate stress or comorbid health conditions that influence mental health. The inclusion of variables such as prophylactic vaccinations and inoculations and “routine chest X-ray suggests a potential relationship between health care utilization and mental health outcomes, possibly reflecting access to health care services. Additionally, neurodevelopmental disorders showed a strong impact on mental health predictions, emphasizing the need to address comorbidities in this population.

Furthermore, the ML models performed better in predicting individual mental health conditions (ie, anxiety or depression) than in predicting both conditions combined. This observation could be attributed to the increased complexity and variability when dealing with multiple conditions simultaneously. Overlapping symptoms may lead to reduced overall performance, and predictive features for one condition may not be as relevant for the other. Focusing on a single condition allows the model to optimize its parameters specifically for that condition and improve performance.

Our study differs from previous research that has primarily relied on questionnaires and neuroimaging data as model outcomes by using EHR data, which, though common, is less frequently applied in adolescent mental health research. For instance, Zhang [25] developed a hybrid convolutional neural network and long short-term memory model trained on a large clinical dataset, including neuroimaging data to predict mental health risks in adolescents. This approach provides valuable insights but focuses on neuroimaging data, which differs from our use of EHR data to address population-level mental health risks. Both approaches contribute uniquely to advancing predictive models in adolescent mental health research. Most studies on adolescent mental health have primarily focused on identifying suicidal attempts or thoughts[26-28], with relatively little focus on addressing conditions such as depression or anxiety in this age group. For example, a study by Sacco et al [29] used EHR data to model suicide risk in the youth but did not specifically address depression or anxiety. Our models achieved AUC values ranging from 0.78 to 0.81, consistent with the 0.8 average reported in a recent review by Nickson et al [30]. Notably, SDoH have not been thoroughly examined in models targeting depression or anxiety in adolescents specifically or in the overall general population.

While our study focuses on data from Florida, USA, it is important to recognize that the prevalence and predictors of depression and anxiety among adolescents vary across different regions and countries. Cultural, social, and health care system differences significantly influence these variations. For instance, a study analyzing data from the Global Burden of Disease Study 2019 found that the prevalence of depression among adolescents and young adults has been rising globally, with notable differences across regions. High-income regions such as North America have experienced significant increases in age-standardized rates for depressive disorders [31]. Additionally, research comparing adolescent mental health in the Nordic countries revealed that socioeconomic inequalities contribute to variations in mental health outcomes. Sweden, for example, exhibited higher rates of mental health problems among adolescents compared to other Nordic countries, potentially due to greater income inequality and higher at-risk-of-poverty rates [32]. These findings suggest that factors such as income inequality, cultural norms, and access to health care services play a crucial role in shaping adolescent mental health outcomes across different regions. Our findings of higher prevalence rates of depression and anxiety among female and White adolescents may align with trends in other high-income countries; however, these patterns can differ in regions with varying socioeconomic structures and cultural contexts.

### Limitations

Our study has several limitations. The data on SDoH and ADI were collected at the community level, which may have obscure significant individual-level differences and reduce the accuracy of our model. Additionally, our models were built using data from a specific group of people and health care system, so it is unclear how well they can generalize to other health care systems with different demographics and SDoH characteristics. The data was gathered at a single point in time; therefore, they do not capture the changing nature of mental health conditions. Additionally, we noticed a higher likelihood of anxiety and depression among White and female adolescents, consistent with the distribution in Florida, which could be influenced by biases in the data or health care system. This pattern warrants further investigation to address potential systemic or reporting biases and to understand unique stressors affecting these groups.

Future research should focus on incorporating longitudinal data to better understand the progression of mental health conditions over time. Additionally, leveraging individual-level SDoH data, such as those derived from clinical notes or surveys, may enhance the predictive accuracy of models. Future studies examining a broader range of demographic and regional settings are also necessary to assess the generalizability of findings. Furthermore, exploring multimodal data sources including genetic, behavioral, and social media data may provide deeper insights into the complex interplay of factors influencing adolescent mental health. Incorporating external validation by testing predictive models on independent datasets from diverse populations and health care systems will be critical for ensuring the reliability and robustness of findings. Finally, targeted studies to understand the intersectionality of race, gender, and socioeconomic status in mental health outcomes are essential to designing equitable and effective interventions.

### Conclusion

This study emphasized the potential of ML in the early detection of depression and anxiety in adolescents using EHRs. It also examined the additive value of community-level SDoH in improving the model’s predictive power. Our models achieved relatively high performance; however, incorporating SDoH did not improve predictive accuracy. This suggests that more detailed, individual-level data may be necessary to enhance predictive power. The implications of this study for clinical practice and decision-making are meaningful; using RWD from EHRs could enable health care providers to more accurately identify at-risk adolescents and intervene earlier, potentially improving mental health outcomes. The ML models that facilitate early detection could allow for prompt mental health interventions, likely enhancing outcomes and lessening the impact of untreated mental health issues. Future studies should focus on integrating individual-level social determinants of health data and validating these models within varied populations to optimize their effectiveness and applicability in clinical and public health environments.

## Supplementary material

10.2196/66665 Multimedia Appendix 1 Supplementary files.

## Abbreviations

ADI: area deprivation index
AUC: area under the curve
EHR: electronic health records
GEOID: geographic identifiers
ICD-10: International Statistical Classification of Disease and Related Health Problems, 10th Revision
ML: machine learning
RWD: real world data
SDoH: social determinants of health
SHAP: shapley additive explanation
UF-IDR: University of Florida integrated data repository
XGBoost: extreme gradient boosting
