# Supplementary material for: Identifying Adolescent Depression and Anxiety Through Real-World Data and Social Determinants of Health: Machine Learning Model Development and Validation
Source: JMIR Ment Health. 2025 Feb 12;12:e66665. doi: 10.2196/66665 (PMC11838812; doi:10.2196/66665)
Supplement: Multimedia Appendix 1 [file mental-v12-e66665-s001.pdf]

Supplementary Material

Table S1. SDoH feature description

**Column**	**Description**	**Universe**	**Category**
PP_less_HS_GED	Percent of population with educational attainment less than Regular high school diploma or GED/alternative credential	Population 25 years and over	Educational Attainment for the Population 25 Years and Over
PP_with_HS_GED	Percent of population with either regular high school diploma or GED/alternative credential	Population 25 years and over	Educational Attainment for the Population 25 Years and Over
PP_bachelors	Percent of population with a bachelor's degree	Population 25 years and over	Educational Attainment for the Population 25 Years and Over
PP_higher_than_bachelors	Percent of population with a higher degree than bachelor's (incl professional,masters or doctorate degree)	Population 25 years and over	Educational Attainment for the Population 25 Years and Over
PP_less_than_bachelors_more_HS_GED	Percent of population with a degree less bachelor's but higher than high school (incl. associate degree)	Population 25 years and over	Educational Attainment for the Population 25 Years and Over
PP_civilian_employed	Percent of population in civilian labour force employed	Population 16 years and over	Employment Status for the Population 16 Years and Over
PP_civilian_unemployed	Percent of population in civilian labour force unemployed	Population 16 years and over	Employment Status for the Population 16 Years and Over
PP_no_comp	Percent of population without a computer	Population in households	Presence of a Computer and Type of Internet Subscription in Household

PP_with_comp	Percent of population with computer	Population in households	Presence of a Computer and Type of Internet Subscription in Household
PP_comp_no_internet	Percent of population with a computer without Internet subscription	Population in households	Presence of a Computer and Type of Internet Subscription in Household
PP_no_internet	Percent of population without Internet subscription	Population in households	Presence of a Computer and Type of Internet Subscription in Household
PP_no_HI	Percent of population without a health insurance	Civilian noninstitutionalized population	Health Insurance Coverage
PP_atleast_one	Percent of population with at least one health insurance	Civilian noninstitutionalized population	Health Insurance Coverage
PP_under_19_no_HI	Percent of population under 19 without a health insurance	Civilian noninstitutionalized population	Health Insurance Coverage
PP_under19_atleast_one	Percent of population under 19 with at least one health insurance	Civilian noninstitutionalized population	Health Insurance Coverage
Median Household Income in the Past 12 Months	Median Household Income in the Past 12 Months	Households	Income
PH_BPL	Percent of households with income in the past 12 months below poverty level	Households	Poverty Status in the Past 12 Months by Household
PH_at_above_PL	Percent of households with income in the past 12 months at or above poverty level	Households	Poverty Status in the Past 12 Months by Household
PP_under_.5	Percent of population for whom income to poverty ratio is less than 0.5	Population for whom poverty status is determined	Ratio of Income to Poverty Level in the Past 12 Months
PP_05_.99	Percent of population for whom income to poverty ratio is between .5 to .99	Population for whom poverty status is determined	Ratio of Income to Poverty Level in the Past 12 Months

PP_1_1.24	Percent of population for whom income to poverty ratio is between 1 to 1.24	Population for whom poverty status is determined	Ratio of Income to Poverty Level in the Past 12 Months
PP_1.25_1.49	Percent of population for whom income to poverty ratio is between 1.25 to 1.49	Population for whom poverty status is determined	Ratio of Income to Poverty Level in the Past 12 Months
PP_1.5_1.84	Percent of population for whom income to poverty ratio is between 1 .5 to 1.84	Population for whom poverty status is determined	Ratio of Income to Poverty Level in the Past 12 Months
PP_1.85_1.99	Percent of population for whom income to poverty ratio is between 1.85 to 1.99	Population for whom poverty status is determined	Ratio of Income to Poverty Level in the Past 12 Months
PP_ABOVE_2	Percent of population for whom income to poverty ratio is above 2	Population for whom poverty status is determined	Ratio of Income to Poverty Level in the Past 12 Months

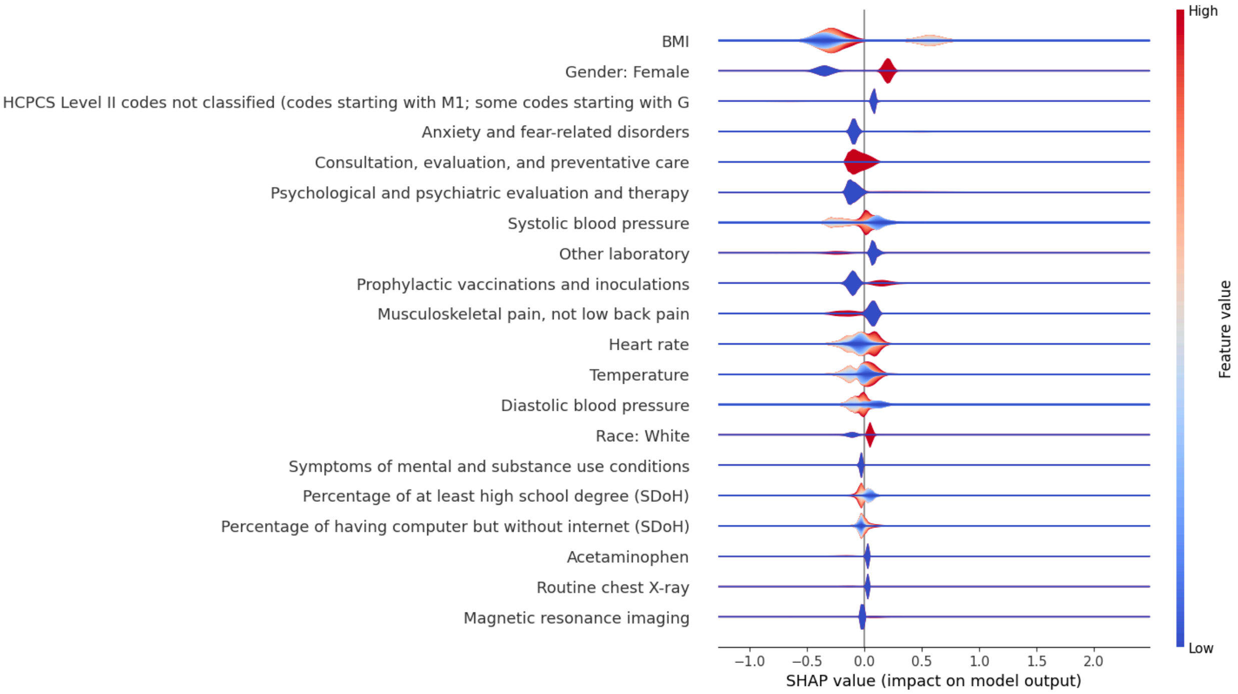

Figure 1. SHAP summary plot for predicting depression by incorporating SDoH features.

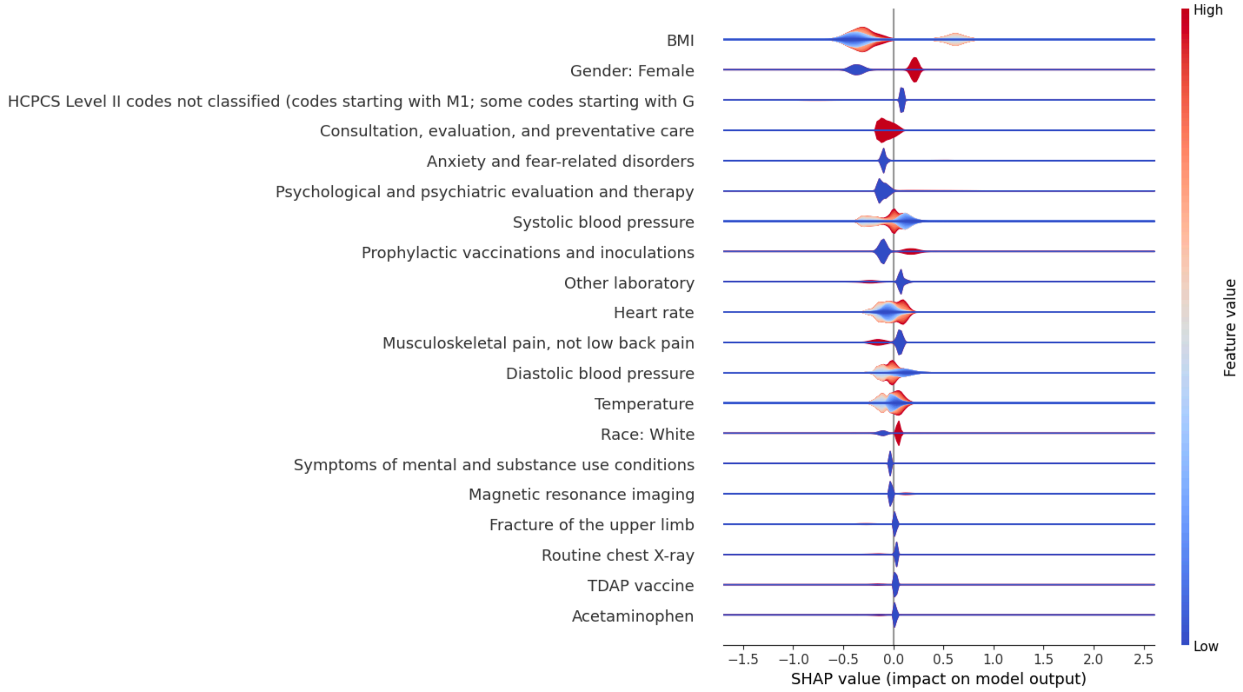

Figure 2. SHAP summary plot for predicting depression by incorporating only ADI.

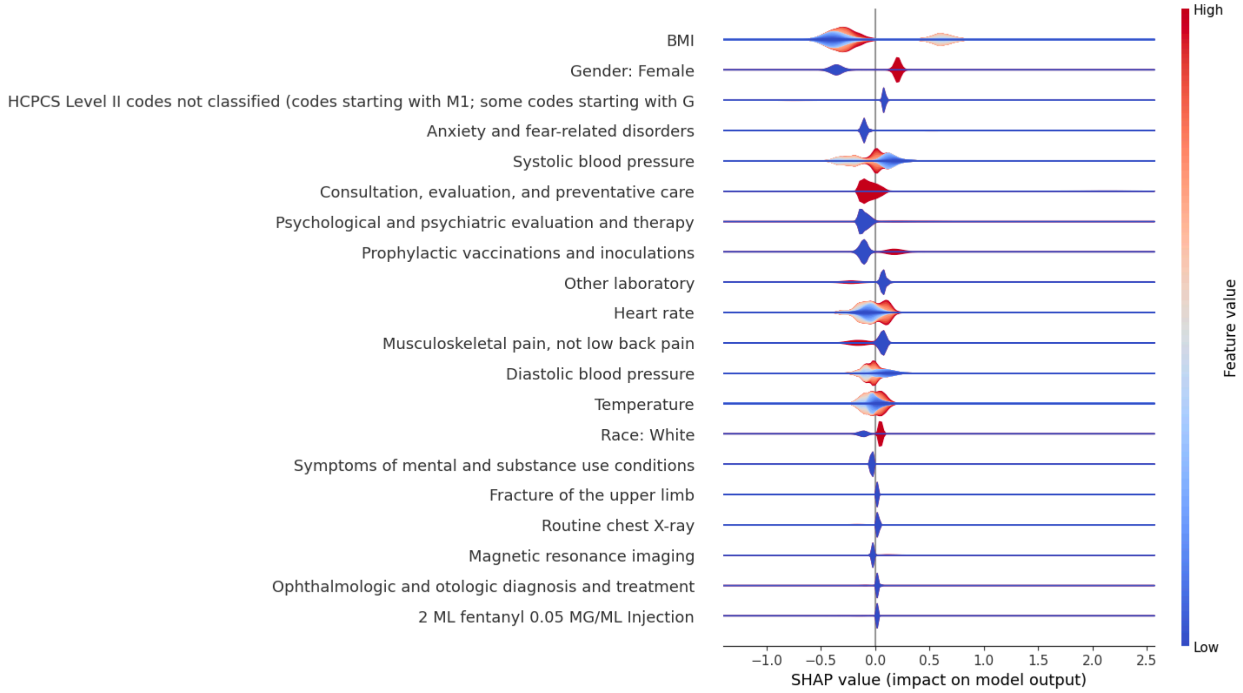

Figure 3. SHAP summary plot for predicting depression without incorporating SDoH features.

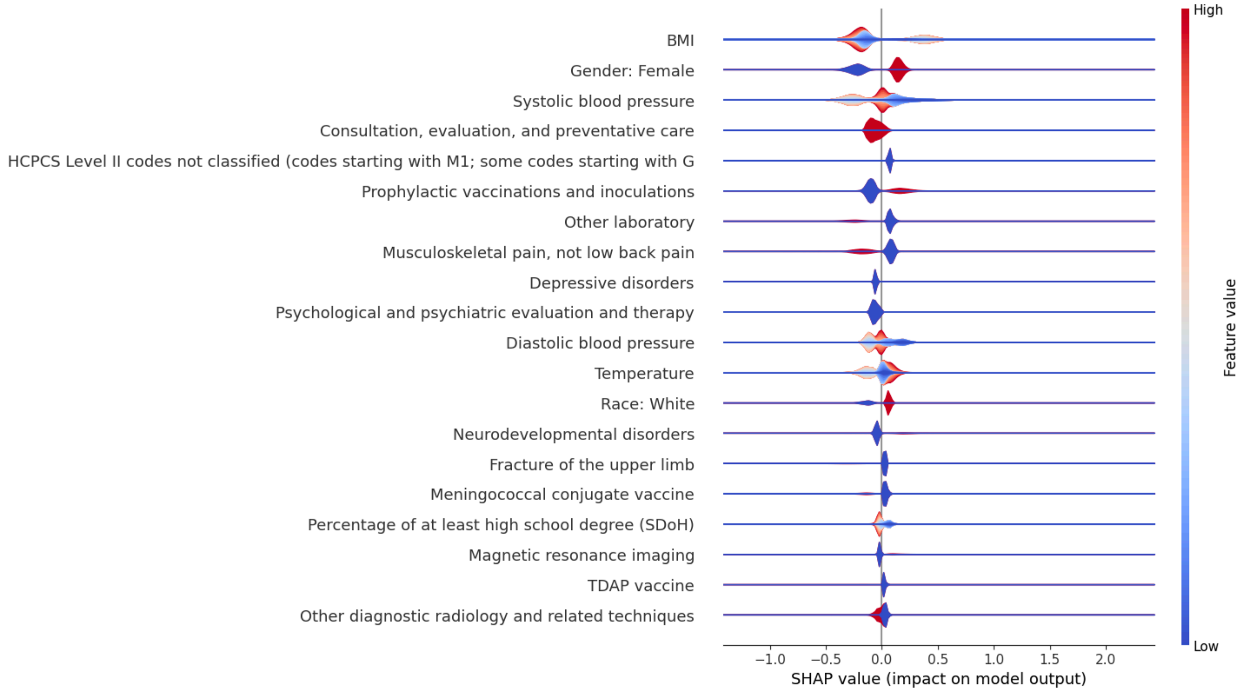

Figure 4. SHAP summary plot for predicting anxiety by incorporating SDoH features.

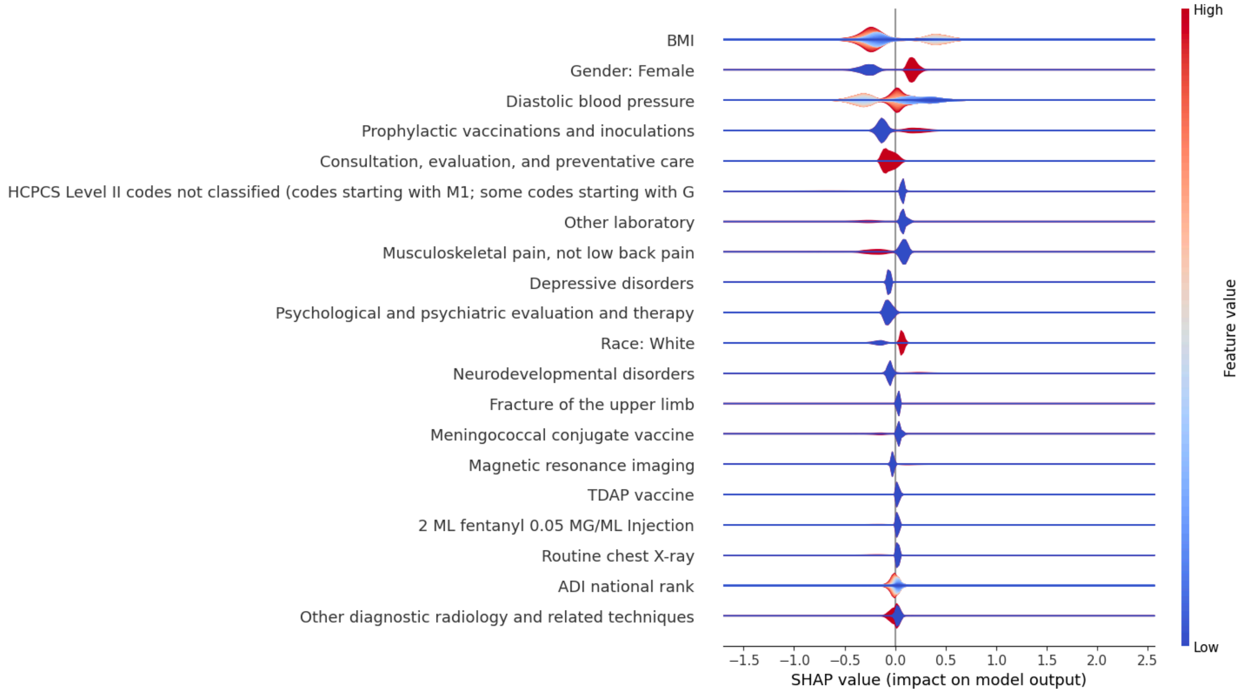

Figure 5. SHAP summary plot for predicting anxiety without incorporating only ADI.

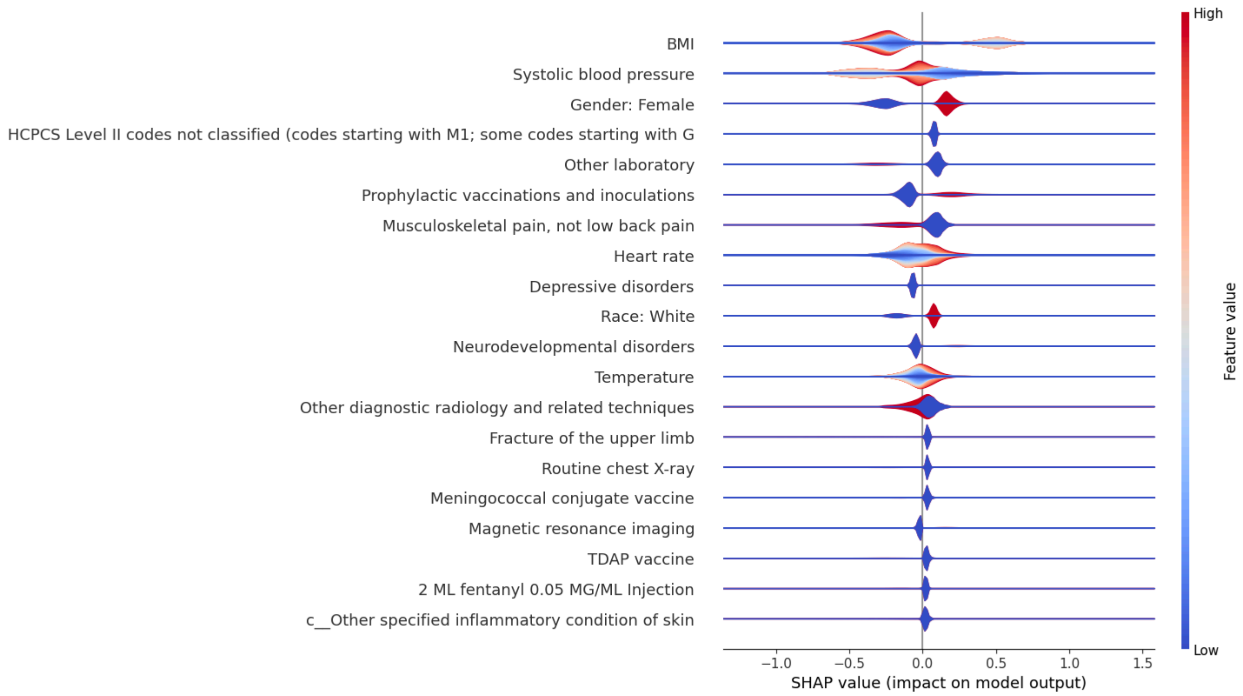

Figure 6. SHAP summary plot for predicting anxiety without incorporating SDoH features.
